# Nasal Adverse Events Associated With Attention-Deficit Hyperactivity Disorder (ADHD) Medications: A Systematic Review, Meta-Analysis, and Implications for the Otolaryngologist

**DOI:** 10.7759/cureus.96878

**Published:** 2025-11-15

**Authors:** Tasnim Kamal, Savan Shah, Umar Ahmed, Hassan Elhassan

**Affiliations:** 1 Otolaryngology, University College Hospital, London, GBR; 2 Otolaryngology, Barking, Havering and Redbridge University Hospitals NHS Trust, London, GBR; 3 Ophthalmology, East Sussex Healthcare NHS Trust, Eastbourne, GBR; 4 Otolaryngology, Homerton University Hospital NHS Foundation Trust, London, GBR

**Keywords:** adult rhinology, attention deficit hyperactivity disorder (adhd), chronic rhinitis treatment, ent pathology, nasopharyngitis, nonallergic rhinitis, non-stimulant, otolaryngology (ent), sinusitis, stimulant medications

## Abstract

This systematic review and meta-analysis aims to determine the incidence of nasopharyngitis and sinusitis associated with stimulant and non-stimulant attention-deficit hyperactivity disorder (ADHD) medications and to assess whether differences exist between drug classes or compared with placebo. Studies published up to 1 August 2025 of adult patients receiving licensed ADHD medications reporting nasal adverse events were included from MEDLINE, Embase, Cochrane, Web of Science, and PsycINFO. Pooled incidence was calculated, and forest plots were constructed for placebo-controlled studies. Subgroup analyses by follow-up duration were performed to explore temporal effects. Risk of bias was assessed, and certainty of evidence was determined using the Grading of Recommendations, Assessment, Development, and Evaluation (GRADE) approach. A total of 18 studies with 5,468 patients met the inclusion criteria, and an additional two articles were used in the subgroup analysis. The pooled incidence of nasopharyngitis was 11.3% (n = 1880) for stimulants and 9.1% (n = 3588) for non-stimulants. Meta-analyses of placebo-controlled studies revealed no significant difference in nasopharyngitis risk between stimulant (RR = 1.09, 95% CI 0.84-1.40) or non-stimulant (RR = 0.91, 95% CI 0.62-1.33) medications and placebo. A Bucher indirect comparison demonstrated no significant differences in nasopharyngitis incidence between drug classes (RR = 1.2, 95% CI 0.76-1.9). Sinusitis occurred in 6.6% (n = 381) and 5.5% (n = 541) of stimulant and non-stimulant users, respectively. Subgroup analysis suggested higher nasopharyngitis rates in stimulant users after 12 months, up to 17% (n = 1018), whereas non-stimulant rates remained consistent over time. Neither stimulant nor non-stimulant ADHD medications were significantly associated with an increased risk of nasopharyngitis or sinusitis compared with placebo. Nevertheless, a subtle trend toward higher incidence with prolonged stimulant use suggests a potential long-term effect. In cases where alternative causes have been excluded, otolaryngologists should remain aware of this possible association. Based on these findings, we propose practical clinical considerations and future research priorities to guide ENT clinicians in optimising the management of ADHD patients with nasal symptoms.

## Introduction and background

Attention-deficit hyperactivity disorder (ADHD) is a common neurodevelopmental disorder that often spans from childhood to adolescence and through adulthood [1]. It is thought to be prevalent in 3% of adults worldwide [2] and is characterised by issues with inattention, hyperactivity, and impulsivity, resulting in debilitating effects on quality of life and day-to-day functioning [3]. There is evidence to suggest a rising diagnosis rate of ADHD. A 2023 study found that the raw number of people with ADHD increased from 72.4 million in 1990 to over 84 million in 2019, a rise of 16.9% globally [4], and a UK study demonstrates a 20-fold increase in ADHD diagnoses and nearly a 50-fold increase in ADHD prescriptions in men between the ages of 18 and 29 [5].

Treatment of ADHD involves pharmacological, behavioural, and combined approaches. Licensed pharmacological therapies can be stratified into stimulant agents (e.g., methylphenidate hydrochloride, lisdexamfetamine dimesylate, amphetamine salts) and non-stimulant agents, further sub-classified as selective noradrenaline reuptake inhibitors (e.g., atomoxetine, viloxazine) and alpha-2 adrenergic receptor agonists (e.g., guanfacine, clonidine) [6].

Stimulant and non-stimulant drugs differ in pharmacodynamics. Stimulants can be either methylphenidate- or amphetamine-based but broadly work by inhibiting both noradrenaline and dopamine transporters, thereby increasing their availability in the presynaptic space and the prefrontal cortex [7-9]. Noradrenaline improves prefrontal cortex function by acting on postsynaptic alpha-2A receptors [10].

Non-stimulant medications include selective noradrenaline reuptake inhibitors such as atomoxetine, which increase noradrenaline in the synapse, thereby improving prefrontal cortex function [6]. Alpha-2 receptor agonists such as guanfacine work post-synaptically, and although the mechanism of action is not completely understood, it is thought to inhibit cAMP production, resulting in closure of HCN channels and improving synaptic transmission [6,11]. Therefore, differential effects on mucosal blood flow, immune modulation, and susceptibility to inflammation are plausible [12].

Modulation of adrenergic signalling also has peripheral effects, including within the nasal mucosa. Adrenergic receptor stimulation influences vascular tone and receptor sensitivity within this region.

Topical decongestants act via this same pathway; however, prolonged use may result in rhinitis medicamentosa, a drug-induced rhinitis characterised by rebound nasal congestion secondary to adrenergic receptor desensitisation and reactive vasodilatation [13]. ADHD medications and topical decongestants may therefore exhibit parallels in their underlying adrenergic mechanisms. However, these parallels are speculative and require further mechanistic study.

Whilst neurological, cardiovascular, and psychiatric side effects of ADHD medications have been widely studied, nasal adverse effects, such as nasopharyngitis and sinusitis, have not been systematically analysed in the literature. By definition, nasopharyngitis and sinusitis are inflammatory processes: nasopharyngitis involves the nasal and pharyngeal mucosa, and sinusitis involves the sinus mucosa. Nasal side effects are reported as adverse events in drug clinical trials. Numerous drug directories, such as the NICE guidelines, the FDA, and Drugs.com, list nasal congestion as a recognised adverse event for both stimulant and non-stimulant medications [14-16].

Increasingly, ADHD patients are presenting to the rhinology clinic with the four cardinal symptoms of nasal disease: nasal obstruction, rhinorrhoea, hyposmia, and facial pressure. These concerns are frequently raised within patient-reported online discussion platforms, reflecting growing patient awareness and symptom burden [17-20]. However, these observations are anecdotal and susceptible to reporting bias.

Identifying and managing the nasal side effects of ADHD medications is an important aspect of optimising ADHD treatment, both for patients and for the otolaryngologist to whom they may present to, as these effects can significantly influence treatment adherence and overall quality of life.

Individual trials and pharmacovigilance reports suggest variable rates of nasal adverse events, but heterogeneous outcome definitions, follow-up durations and comparator groups leave uncertainty for clinicians and guideline writers. To address this gap, we performed a systematic review (and, where appropriate, a meta-analysis) with the following aims: (1) to estimate the pooled incidence of nasopharyngitis and sinusitis amongst adults treated with licensed stimulant versus non-stimulant ADHD medications; (2) to compare risk versus placebo in randomised controlled trials; and (3) to explore whether incidence varies with follow-up duration.

## Review

Methods

Outcomes

We performed a systematic review of peer-reviewed published studies of ADHD medications reporting nasal complications, such as nasopharyngitis and sinusitis, in accordance with PRISMA (Preferred Reporting Items for Systematic Reviews and Meta-Analyses) guidelines. Our primary outcome was the incidence of nasal obstruction/congestion, nasopharyngitis, rhinitis, sinusitis, or other nasal symptoms with stimulant vs. non-stimulant ADHD drugs.

Inclusion and Exclusion Criteria

Our inclusion criteria were studies that reported nasal symptoms as adverse events in patients treated with a licensed ADHD medication. We included studies of patients aged 18 or over with a formal diagnosis of ADHD. We also included randomised controlled trials, prospective cohorts, and observational studies.

Exclusion criteria were paediatric-only studies, case reports or series with fewer than 10 patients, studies without drug-specific nasal outcome data, studies in which outcomes of different medication types could not be differentiated, and animal studies. We also excluded single-dose pharmacokinetic or tolerability studies, as our focus was on adverse events during clinically relevant treatment durations. Articles with broad subheadings of adverse events, for example, "URTI", were excluded if they did not specify the incidence of specific nasal adverse events.

Search Strategy

Searches of eligible English language articles were performed in the following databases: MEDLINE, Embase, Cochrane Database of Systematic Reviews, Cochrane Central Register of Controlled Trials (CENTRAL), Web of Science, and PsycINFO. ClinicalTrials.gov, the EU trials register, and grey literature were also sourced. The search was conducted on 1 August 2025 and included studies published between 1 January 1980 and the search date.

Search terms were split into three arms and included (1) "attention deficit hyperactivity disorder" and variations; (2) drug classes: "methylphenidate", "lisdexamfetamine", "atomoxetine", "guanfacine", "clonidine", and variations, including brand names; (3) nasal symptoms: "nasal obstruction", "nasopharyngitis", "sinusitis", and "nasal*". The complete search strategy can be found in Supplementary Appendices A-C.

A reference review of the included studies was further performed. Two review authors (T.R.K. and S.S.) then screened titles and abstracts for relevance to retrieve the full texts of potentially relevant studies. Any disagreements regarding the full-text articles were discussed until the review authors reached a consensus. If required, correspondence with investigators was undertaken to provide clarification on studies and definitively determine eligibility.

Data were extracted independently by two review authors (T.R.K. and S.S.) and inspected for discrepancies. There were no non-English-language publications captured by the search.

Where there was suspicion that patients had been included in more than one study, efforts were made to contact the authors to determine the extent of overlap. In cases where this was not possible, we included the original trial report in the primary pooled analysis to avoid double-counting. Subsequently, post-hoc analyses were summarised narratively and included in sensitivity analyses, where they replaced the parent report. Any further information required from the original author was requested by written correspondence, and any relevant information obtained in this manner was included in the review.

Data Extraction

From each article, we extracted data available on the following: country of origin, study design, drug name, drug class, drug mechanism of action, drug dose, comparator (for example, placebo), nasal adverse event type, incidence of nasal symptoms, sample size, follow-up duration, sources of bias and/or conflict.

Data Analysis

Data on our primary outcomes were reported in three ways. Firstly, summary tables of included studies were presented, along with a pooled analysis of the incidence of nasal side effects for stimulant vs. non-stimulant medications. Secondly, given the expected clinical and methodological heterogeneity across trials (different drugs, doses, and follow-up durations), a random-effects model was employed to provide a more conservative estimate of pooled effects. Study weights were assigned using the inverse-variance method, which accounts for both the study sample size and the precision of its effect estimate. A forest plot was generated in Microsoft Excel (Microsoft Corporation, Redmond, Washington) to determine statistical significance between ADHD medications and placebo for the incidence of nasopharyngitis in studies that included placebo comparators. Visual assessment of forest plots was used to assess heterogeneity. As the included studies were similar in design and population, indirect comparison between stimulant and non-stimulant medications was performed using the Bucher method, with placebo as the common comparator. Finally, studies were stratified and presented in a table according to length of follow-up to determine whether this affected the incidence of nasal adverse events.

Two additional articles, Buitelaar et al. (2011) and Huss et al. (2014) [21,22], were not included in the pooled analysis but were accounted for through sensitivity analysis and in our follow-up subgroup analysis. These studies were not included in the original pooled analysis to avoid double-counting, as they were an extension of previous studies and therefore represented a secondary reclassification of the same population.

Risk of Bias Assessment

Risk of bias in the included studies was assessed systematically using the GRADE (Grading of Recommendations, Assessment, Development, and Evaluation) tool.

Results

Study Selection

**Figure 1:**
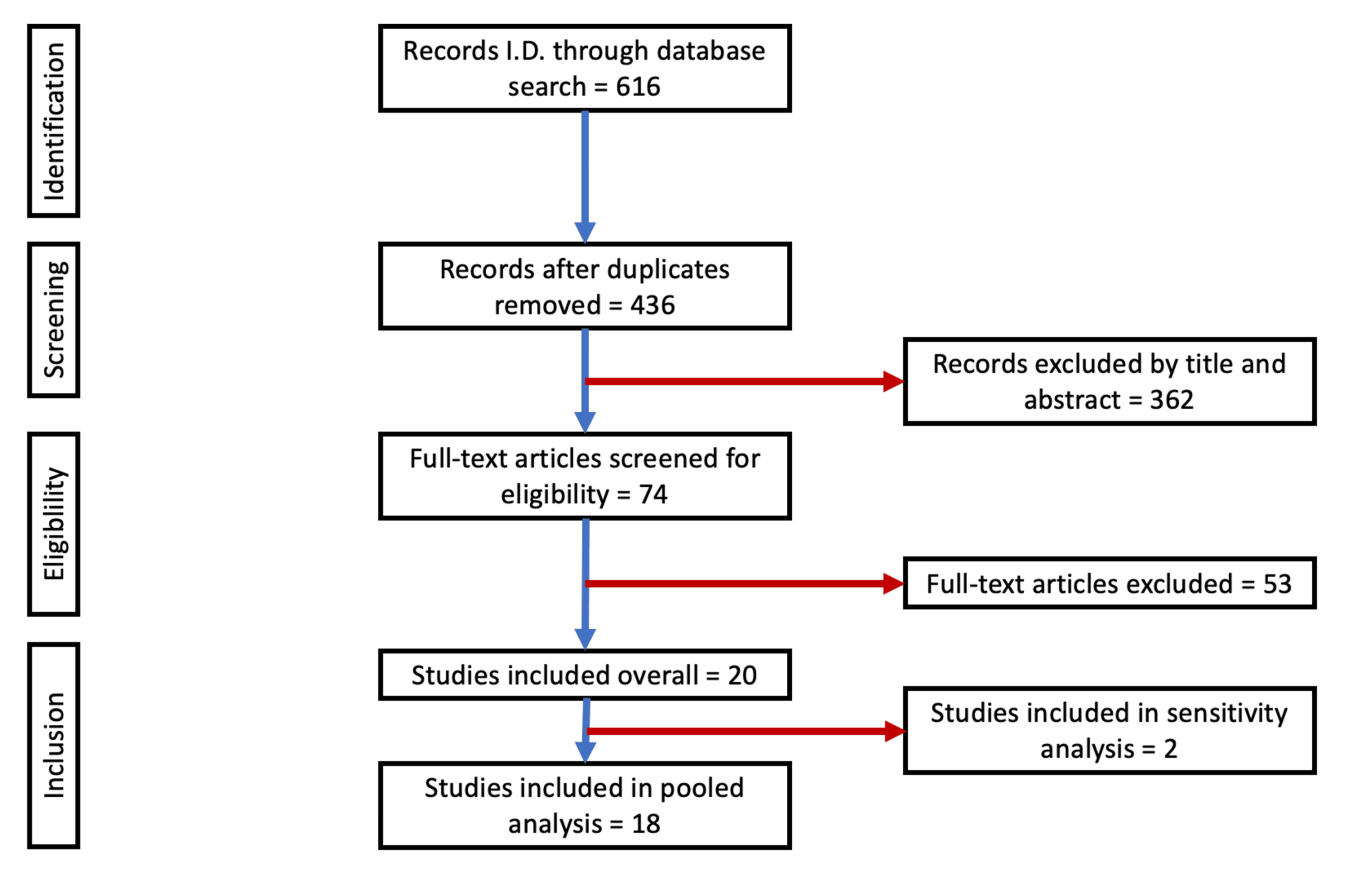
PRISMA flowchart depicting the process of article selection. PRISMA: Preferred Reporting Items for Systematic Reviews and Meta-Analyses

Our most recent search, conducted on 4 August 2025, identified 616 studies. After removing duplicates, 436 records remained. Of these, 74 articles were read and assessed for eligibility after screening them by title and abstract. A total of 20 full-text articles were found to be eligible for inclusion. Of those, 18 were eligible for pooled analysis of nasal symptom rates, with 1880 and 3588 patients treated with stimulant and non-stimulant medications, respectively. Two studies, Buitelaar et al. (2011) [21] and Huss et al. (2014) [22], were included as part of a sensitivity analysis due to overlapping data. A PRISMA flow diagram was used to illustrate the study screening process (Figure 1).

Incidence of Nasopharyngitis - Stimulant Medications

Table 1 depicts a summary of seven studies that report the incidence of nasopharyngitis for stimulant ADHD medications, including various study characteristics [23-29]. The studies were a mixture of randomised controlled trials and open-label extensions, studying the following stimulant medications: OROS-MPH, MPH-LA, and LDX. Pooled analysis of studies (avoiding duplication) showed an overall incidence of nasopharyngitis was 11.3% (n = 1880) for stimulant medications. This ranged from 5.1% (n = 79) reported by Adler et al. (2013) [28] for LDX to as high as 27.3% (n = 216) as reported by Ginsberg et al. (2014) [26] for MPH-LA.

**Table 1:** Characteristics of the seven included studies of stimulant medications with reported outcomes of nasopharyngitis, including pooled incidence. *Sum of "overall" dose incidence, not individual dose incidence Abbreviations: OROS-MPH = osmotic release oral system-methylphenidate; MPH-LA = long-acting methylphenidate formulation; LDX = lisdexamfetamine dimesylate; RCT = randomised controlled trial

Study	Country	Study Design	Drug	Dose (mg/day)	Comparator	F/u Duration (Weeks)	Events (n)	Sample Size (N)	Incidence (%)
Buitelaar et al. 2009 [23]	Europe	open label	OROS-MPH	mean = 47.5	nil	7	21	370	5.7
Casas et al. 2013 [24]	Europe	RCT	OROS-MPH	54	Placebo	13	13	89	14.6
				72			11	92	12
Takahashi et al. 2014 [25]	Japan	RCT	OROS-MPH	18–72	Placebo	8	24	143	16.8
Ginsberg et al. 2014 [26]	6 countries	open label	MPH-LA	40–60	Placebo	52	59	216	27.3
Huss et al. 2013 [27]	9 countries	RCT	MPH-LA	overall	Placebo	40	54	542	10
				40			22	180	12.2
				60			15	181	8.3
				80			17	181	9.4
Adler et al. 2013 [28]	USA	RCT	LDX	30–70	Placebo	10	4	79	5.1
Weisler et al. 2009 [29]		open label	LDX	All doses	nil	52	26	349	7.4
				30			6	349	1.7
				50			11	323	3.4
				70			13	238	5.5
Pooled incidence*							212	1880	11.3

We also undertook two sensitivity analyses for nasopharyngitis with stimulant medications, substituting data from two longer-term follow-up studies: Buitelaar et al. (2011) [21] and Huss et al. (2014) [22].

Huss's original randomised controlled trial [27] reported nasopharyngitis in 10% (n = 542) of participants overall and was included in the primary analysis. A subsequent extension study by the same authors reported a re-analysed rate of 19.1% (n = 298) in an overlapping cohort [22]. In the sensitivity analysis, substituting the original study [27] with the extension study [22] increased the pooled incidence from 11.3% (n = 1880) to 13.1% (n = 1636). Similarly, sensitivity analysis substituting Buitelaar et al. (2009) [23], which had a 5.7% (n = 370) incidence of nasopharyngitis, with Buitelaar et al. (2011) [21], with a 20% (n = 155) incidence, increased nasopharyngitis incidence from 11.3% (n = 1880) to 13.3% (n = 1665) in the sensitivity analysis.

**Figure 2:**
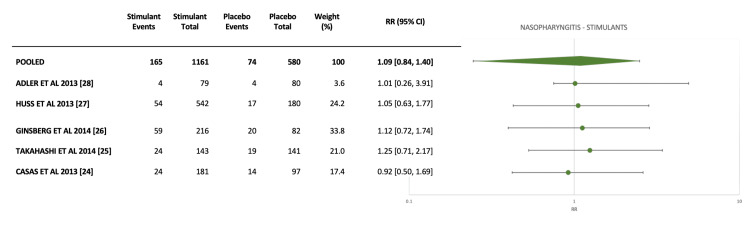
Forest plot including five studies that reported placebo-controlled outcomes for nasopharyngitis with stimulant medications.

We conducted a meta-analysis of nasopharyngitis incidence with stimulant medications, including five studies with placebo controls [24-28]. Four of these were RCTs, and one was an open-label extension study (Figure 2). The pooled relative risk (RR) for nasopharyngitis with stimulant use was 1.09 (95% CI: 0.84-1.4), demonstrating no significant differences between stimulant medication and placebo. Individual study estimates were directionally consistent, with all confidence intervals crossing 1, and no outliers were identified.

Incidence of Nasopharyngitis - Non-stimulant Medications

Table 2 summarises nine studies reporting the incidence of nasopharyngitis with non-stimulant ADHD medications [30-38]. The studies were a mixture of randomised controlled trials and open-label extensions that studied the following non-stimulant medications: atomoxetine and guanfacine. The pooled analysis of studies that avoided duplication indicated that the overall incidence of nasopharyngitis with non-stimulant medications was 9.1% (n = 3588). Incidence ranged from 4% (n = 243) for atomoxetine (Adler et al. 2009) [30] to 35.6% (n = 45), again for atomoxetine (Takahashi et al. 2011) [36].

**Table 2:** Characteristics of the nine included studies of non-stimulant medications with reported outcomes of nasopharyngitis, including pooled incidence. Abbreviation: RCT = randomised controlled trial

Study	Country	Study Design	Drug	Dose (mg/day)	Comparator	F/u Duration (Weeks)	Events (n)	Sample Size (N)	Incidence (%)
Adler et al. 2009 [30]	USA	RCT	Atomoxetine	Up to 100 mg. Depend on tolerability	Placebo	24	9	243	4
Adler et al. 2005 [31]	USA + Canada	Open label	Atomoxetine	25/40/60	nil	156	36	382	9.4
Camporeale et al. 2013 [32]	18 countries: Asia, North America	RCT	Atomoxetine	40–100	Placebo	25	18	266	6.8
Hirata et al. 2013 [33]	Japan	Open label	Atomoxetine	40–120	nil	Overall	59	233	25.3
						0–12	23	233	9.9
						12–24	21	196	10.7
						24–36	11	167	6.6
						36–48	4	149	2.7
						84+	0	81	0
Iwanami et al. 2020 (early) [34]	Japan	RCT	Guanfacine	2, 4, 6	Placebo	13	19	101	18.8
Iwanami et al. 2020 [35]	Japan	Open label	Guanfacine	2, 4, 6	nil	50	14	41	34.1
Takahashi et al. 2011 [36]	Japan	Open label	Atomoxetine	40–120	nil	8	16	45	35.6
Tanaka et al. 2016 [37]	Europe, US, Latin America	Retrospective analysis of prev. RCT	Atomoxetine	Variable (40–100)	Placebo	25	18	266	6.8
Upadhyaya et al. 2015 [38]	18 countries	Retrospective analysis of prev. RCT	Atomoxetine	Variable (40–100)	nil	49	137	2011	6.8
Pooled incidence							326	3588	9.1

It should be noted that both Iwanami's studies were included, as there was a clear delineation between the participants in each study, which allowed us to avoid overlap when pooling [34,35].

**Figure 3:**
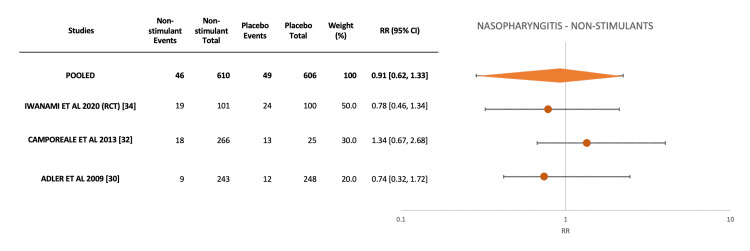
Forest plot including four studies that reported placebo-controlled outcomes for nasopharyngitis with non-stimulant medications.

For meta-analysis of nasopharyngitis incidence with non-stimulant medications, three studies were included [30,32,34]. A pooled RR of 0.91 (95% CI: 0.62-1.33) again demonstrates no significant differences between non-stimulant medication and placebo. This was also noted in individual studies, with all confidence intervals crossing 1, and no outliers were identified (Figure 3).

A Bucher indirect comparison was performed using placebo as the common comparator to estimate the RR of nasopharyngitis between stimulant and non-stimulant ADHD medications. The analysis demonstrated no statistically significant difference between drug classes (RR = 1.2, 95% CI = 0.76, 1.9), suggesting comparable nasal adverse event profiles.

Incidence of Sinusitis

Table 3 depicts a summary of studies that report the incidence of sinusitis for stimulant ADHD medications, including various study characteristics [29,39]. The following stimulant medications were included: Osmotic Release Oral System-Methylphenidate (OROS-MPH) and Lisdexamfetamine dimesylate (LDX).

**Table 3:** Characteristics of the two included studies of stimulant medications with reported outcomes of sinusitis, with pooled incidence. Abbreviations: OROS-MPH = osmotic release oral system-methylphenidate; LDX = lisdexamfetamine dimesylate

Study	Country	Study Design	Drug	Dose (mg/day)	Comparator	F/u Duration (Weeks)	Events (n)	Sample Size (N)	Incidence (%)
Fallu et al. 2006 [39]		Open label	OROS-MPH	Max 72	nil	5.4	2	32	6
Weisler et al. 2009 [29]	USA	Open label	LDX	All doses	nil	52	23	349	6.6
				30			2	349	0.6
				50			10	323	3.1
				70			12	238	5
Pooled incidence							25	381	6.6

Table 4 depicts a summary of studies that report the incidence of sinusitis for non-stimulant ADHD medications, including various study characteristics [31,40]. The non-stimulant medications included are atomoxetine and viloxazine extended release (ER).

**Table 4:** Characteristics of the two included studies of non-stimulant medications with reported outcomes of sinusitis, with pooled incidence. Abbreviation: ER = extended release

Study	Country	Study Design	Drug	Dose (mg/day)	Comparator	F/u Duration (Weeks)	Events (n)	Sample Size (N)	Incidence (%)
Adler et al. 2005 [31]	USA + Canada	RCT + open label	Atomoxetine	25/40/60	nil	156	26	382	6.8
Childress et al. 2024 [40]		Open-label extension to double-blind	Viloxazine ER	200-600	Placebo	38 ± 36.4 days	4	159	2.5
Pooled incidence							30	541	5.5

A pooled analysis of studies (avoiding duplication) showed an overall incidence of sinusitis of 6.6% (n = 381) and 5.5% (n = 541) for stimulants and non-stimulants, respectively. No sensitivity analysis was required due to the lack of overlapping studies, and meta-analysis was not possible due to the low number of placebo-controlled studies.

After exclusion, no other rhinological symptoms were reported in the studies, such as facial pain, post-nasal drip, or rhinitis.

Studies With Longer-Term Follow-Up

Table 5 stratifies the 18 articles from the pooled analysis into follow-up periods of less than six months, 6-12 months, and more than 12 months [23-40].

**Table 5:** Complication rates stratified by follow-up period: less than or equal to six months, between six and 12 months, and equal to or over 12 months *Substitution with longer-term extension studies – Buitelaar et al. 2009 [23] with Buitelaar et al. 2011 [21] and Huss et al. 2013 [27] with Huss et al. 2014 [22] i) Buitelaar et al. 2009 [23], Casas et al. 2013 [24], Takahashi et al. 2014 [25], Adler et al. 2013 [28] ii) Huss et al. 2013 [27] iii) Ginsberg et al. 2014 [26], Weisler et al. 2009 [29] iv) Adler et al. 2009 [30], Camporeale et al. 2013 [32], Iwanami et al. 2020 [34], Takahashi et al. 2011 [36], Tanaka et al. 2017 [37], Hirata et al. 2014 [33] v) Hirata et al. 2014 [33], Iwanami et al. 2020 [35], Upadhyaya et al. 2015 [38] vi) Adler et al. 2005 [31], Hirata et al. 2014 [33] vii) Fallu et al. 2006 [39] viii) Weisler et al. 2009 [29] ix) Childress et al. 2024 [40] x) Adler et al. 2005 [31] xi) Casas et al. 2013 [24], Takahashi et al. 2014 [25], Adler et al. 2013 [28] xii) Buitelaar et al. 2011 [21], Ginsberg et al. 2014 [26], Weisler et al. 2009 [29], Huss et al. 2014 [22]

Follow-Up Duration (Months)	≤6	>6 to <12	≥12
Nasopharyngitis incidence (%)			
Stimulants (Buitelaar 2009 + Huss 2013)	9.4 (n=773)^i^	10 (n=542)^ii^	15 (n=565)^iii^
Stimulants (Buitelaar 2011 + Huss 2014)*	12.9 (n=403)^xi^	0	17 (n=1018)^xii^
Non-stimulants	9.2 (n=1350)^iv^	7.0 (n=2368)^v^	7.8 (n=463)^vi^
Sinusitis incidence (%)			
Stimulants	6 (n=32)^vii^	-	6.6 (n=349)^viii^
Non-stimulants	-	2.5 (n=159)^ix^	(n=382)^x^

For stimulant medications, four studies demonstrated a 9.4% (n = 733) pooled nasopharyngitis incidence for a less than six-month follow-up period [23-25,28], which increased marginally to 10% (n = 542) with one study that had a six- to 12-month follow-up [27] and subsequently increased to 15% (n = 565) for two studies with a follow-up period of at least 12 months [26,29].

Substituting data from Buitelaar et al. 2009 [23] with Buitelaar et al. 2011 [21] and Huss et al. 2013 [27] with Huss et al. 2014 [22] (longer-term extension studies) increased these overall percentages in all categories to 12.9% (n = 403) for studies with less than six months follow-up and 17% (n = 1018) for studies with greater than 12 months follow-up. There were no studies with a six- to 12-month follow-up in this re-analysis.

For non-stimulant medications, nasopharyngitis incidence was 9.2% (n = 1350, six studies) [30,32-34,36,37], 7% (n = 2368, three studies) [33,35,38], and 7.8% (n = 463, two studies) [31,33] for less than six months, 6-12 months, and 12 months or more follow-up periods, respectively.

The incidence of sinusitis was less frequently reported. For stimulant ADHD medications, one study [39] with a 38-day follow-up exhibited a 6% (n = 32) incidence, whereas another study [29] with a 12-month follow-up demonstrated a 6.6% (n = 349) incidence. Conversely, for non-stimulant ADHD medications, one study with a mean follow-up of 265 days (between six and 12 months) had a 2.5% (n = 159) incidence of sinusitis [40], and this increased to 6.8% (n = 382) for a study with a three-year follow-up [31].

Discussion

Summary of Findings

Overall, our findings point towards stimulant and non-stimulant ADHD medications having comparable incidences of both nasopharyngitis and sinusitis. Our meta-analyses of available placebo-controlled studies demonstrated no significant differences in nasopharyngitis incidence between active drug and placebo for both stimulant and non-stimulant medications (Figures 2, 3, respectively), with risk ratios clustering together and confidence intervals overlapping 1. Other nasal adverse events were rarely reported in the studies.

Controlling for variable follow-up periods by stratifying studies by follow-up duration, however, may suggest an increase in nasopharyngitis incidence with stimulant medications in the longer term, particularly beyond 12 months (see Table 5). Non-stimulant agents, however, have a similar incidence regardless of follow-up period.

Future trials with extended follow-up and standardised adverse event reporting could help clarify whether this trend represents a genuine pharmacological effect or background variation.

Interpretation and Biological Plausibility

Rhinologists often receive referrals for what is presumed to be drug-induced rhinitis, presenting in patients taking ADHD medications. Adverse nasal side effects are also anecdotally discussed in Internet forums such as "Reddit" and "Totally ADD", particularly regarding stimulant medications such as Adderall and Vyvanse [17-20]. Therefore, the question of whether this observation is supported in the literature is highly clinically relevant.

It is plausible that stimulant ADHD medications result in complications such as nasopharyngitis, sinusitis, and other nasal symptoms. Stimulant agents such as methylphenidate and lisdexamfetamine induce sympathetic activation and can therefore result in nasal mucosal vasoconstriction [41]. This may induce an initial decongestive effect in the nasal mucosa; after all, historically, the topical amphetamine "Benzedrine" was used as a nasal decongestant [42]. However, with chronic exposure, patients may be predisposed to rhinitis medicamentosa, resulting in mucosal dryness, reduced ciliary clearance, or secondary infections [12]. This may explain our findings of a modest increase in nasopharyngitis incidence for stimulant medications in the long term. However, note that rhinitis medicamentosa remains incompletely understood, and physiological studies are conducted for local/topical administration; limited data are available for systemic administration.

Conversely, non-stimulants act through noradrenergic and alpha-2 adrenergic pathways. For example, alpha-2 agonists, such as guanfacine and clonidine, are sympatholytic drugs which act centrally to reduce the release of norepinephrine, leading to vasodilation and increased vascular permeability in nasal blood vessels [6].

As nasal decongestants, stimulant and non-stimulant ADHD medications converge on a common mechanism: direct and indirect stimulation of alpha-receptors. It is plausible that they exert variable vasoconstrictive and vasodilative effects on the nasal vasculature.

However, as the majority of our findings suggest no significant association between stimulant versus non-stimulant medications and nasal adverse events, it is also plausible that the incidence of nasal adverse events is instead reflective of a greater background incidence of nasopharyngitis and sinusitis in adult ADHD populations.

This hypothesis is supported by numerous studies that propose a link between ADHD and allergic rhinitis, albeit the majority of these studies are based on a paediatric population or are mechanistic studies [43-45]. There is thought to be a close relationship between the pathophysiology of allergy and neurodevelopmental diseases, with overlapping inflammatory cytokine response and immune cell dysregulation [45]. If this is the case, ADHD medications might unmask a predisposed inflammatory phenotype in susceptible individuals rather than induce de novo pathology.

Agreements and Disagreements With Other Literature

There are limited studies on nasal adverse events as a primary outcome when it comes to stimulant and non-stimulant ADHD medications. Where prior meta-analyses have focused on psychiatric or cardiovascular outcomes, ours provides the first class-specific synthesis for ENT outcomes.

Quality of the Included Studies and Limitations of Our Study

The risk of bias GRADE table highlights the quality of different types of studies included (Table 6). Our included studies are a mixture of higher-quality randomised, double-blind, placebo-controlled trials and open-label extensions of randomised controlled trials. However, our study is not without limitations.

**Table 6:** Characteristics and assessment of bias of the included studies Abbreviations: RCT = randomised controlled trials; GRADE = Grading of Recommendations Assessment, Development, and Evaluation

Included Studies	Characteristics of Studies	Assessment of Bias
RCTs		
Adler et al. 2009 [30], Camporeale et al. 2013 [32], Iwanami et al. 2020 [34], Casas et al. 2013 [24], Takahashi et al. 2014 [25], Huss et al. 2013 [27], Adler et al. 2013 [28]	Placebo-controlled, randomised controlled studies reporting specific adverse events	Most studies double-blinded
	Shorter follow-up, but sufficient to determine the presence or absence of nasal adverse events at the time	Moderate inconsistency of outcomes between studies
		Moderate publication bias
		Funding bias (pharmaceutical)
		GRADE certainty of individual studies moderate to high
Open-label extension studies with placebo		
Ginsberg et al. 2014 [26]	Prospective studies reporting specific adverse events	Placebo-control reduces bias
	Outcomes were reported consistently across all patients in the study	Funding bias (pharmaceutical)
	Longer follow-up sufficient to determine the presence or absence of nasal adverse events at the time	GRADE certainty of individual studies moderate
Open-label extension studies		
Buitelaar et al. 2009 [23], Weisler et al. 2009 [29], Adler et al. 2005 [31], Hirata et al. 2014 [33], Iwanami et al. 2020 [35], Takahashi et al. 2011 [36]	Prospective studies reporting specific adverse events	No controls increases bias
		Moderate inconsistency of outcomes between studies
	Outcomes were reported consistently across all patients in the study	Moderate publication bias
	Longer follow-up sufficient to determine the presence or absence of nasal adverse events at the time	Funding bias (pharmaceutical)
		GRADE certainty of individual studies is low to moderate
Retrospective analyses of previous RCTs		
Tanaka et al. 2017 [37], Upadhyaya et al. 2015 [38]	Retrospective studies reporting specific adverse events	Low inconsistency of outcomes between studies
	Outcomes were reported consistently across all patients in the study	Moderate publication bias
	Longer follow-up sufficient to determine the presence or absence of nasal adverse events at the time	Funding bias (pharmaceutical)
		GRADE certainty of individual studies is low to moderate

Firstly, the definitions of outcomes varied across trials; many listed "nasopharyngitis" or "sinusitis" as spontaneously reported adverse events without standardised diagnostic criteria, raising the possibility of misclassification. Secondly, trial durations were often short (weeks to months), limiting capture of less frequent or delayed events. Also, the follow-up durations across the studies included in both the pooled and meta-analyses are not equivalent. We tried to account for this with a subgroup analysis of variable follow-up periods. Thirdly, many studies were industry-sponsored and may be susceptible to selective adverse event reporting and reporting bias. Fourthly, an assumption was also made that all data collected from a given study were sufficiently homogenous to allow data pooling. This could be a flawed assumption, seeing that our patient demographic varied, for example, by study location (Europe vs. Asia vs. America), variable dosages were often optimised to specific patients, and there were diverse patient co-morbidities. Adults with comorbid psychiatric disease and significant cardiovascular disease were excluded from certain studies, potentially limiting the ability to generalise these results to the broader adult population with ADHD [39].

Future Directions

Future research should focus on prospective studies with pre- and post-treatment nasal assessments, incorporating objective measures such as endoscopic scoring, CT sinus scans, and inflammatory biomarker profiling. Integration of ENT outcome measures into ADHD drug trials could clarify whether nasal adverse events are pharmacologically mediated or reflect underlying neuroimmune overlap between ADHD and allergic airway disease. These ENT outcomes should be prespecified using standard diagnostic criteria (e.g., EPOS criteria for sinusitis).

Additionally, in the included studies, whilst both drug classes are compared to placebo, head-to-head trials and ultimately a direct or network meta-analysis would be needed to confirm whether any true difference exists between the two drug types.

Clinical Perspectives

**Figure 4:**
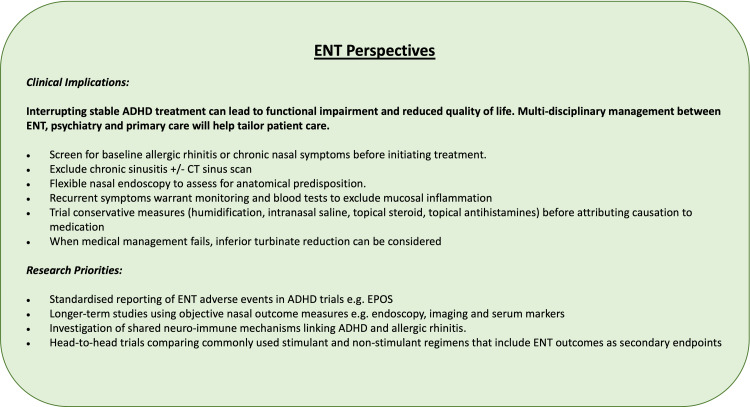
ENT perspectives. Image created by the authors.

Accounting for our findings and the wider literature, we have created "ENT Perspectives" to guide rhinologists who encounter patients with ADHD in the clinic (Figure 4).

## Conclusions

In summary, we have provided the first systematic review comparing rhinological outcomes between stimulant and non-stimulant ADHD therapies. Although neither stimulant nor non-stimulant ADHD medications were significantly associated with an increased risk of nasopharyngitis or sinusitis compared with placebo, a trend towards a higher incidence with prolonged stimulant use suggests a potential long-term effect. In cases where alternative causes have been excluded, otolaryngologists should consider this possible association in their clinical practice. Based on these findings, we propose practical clinical considerations and future research priorities to guide ENT clinicians in optimising the management of ADHD patients presenting with nasal symptoms.

## Appendices

**Table 7:** Supplementary Appendix A - EMBASE search strategy.

1.	*attention deficit hyperactivity disorder/	
2.	adhd.ab,ti.	
3.	Attention Deficit Hyperactivity Disorder.ab,ti.	
4.	Attention Deficit Disorder with Hyperactivity.ab,ti.	
5.	Attention Deficit Disorder.ab,ti.	
6.	Hyperkinetic Syndrome.ab,ti.	
7.	1 or 2 or 3 or 4 or 5 or 6	
8.	*central stimulant agent/	
9.	*methylphenidate/	
10.	methylphenidate.ab,ti.	
11.	Ritalin.ab,ti.	
12.	Concerta.ab,ti.	
13.	Medikinet.ab,ti.	
14.	Equasym.ab,ti.	
15.	Quillivant.ab,ti.	
16.	Daytrana.ab,ti.	
17.	Metadate.ab,ti.	
18.	*amphetamine/	
19.	"amphetamine*".ab,ti.	
20.	*amphetamine plus dexamphetamine/	
21.	Adderall.ab,ti.	
22.	Evekeo.ab,ti.	
23.	Mydayis.ab,ti.	
24.	*dexamphetamine/	
25.	Dextroamphetamine.ab,ti.	
26.	"dexamphetamine*".ab,ti.	
27.	Dexedrine.ab,ti.	
28.	*lisdexamfetamine/	
29.	Lisdexamphetamine.ab,ti.	
30.	Vyvanse.ab,ti.	
31.	*atomoxetine/	
32.	Atomoxetine Hydrochloride.ab,ti.	
33.	atomoxetine.ab,ti.	
34.	Strattera.ab,ti.	
35.	*guanfacine/	
36.	Guanfacine.ab,ti.	
37.	Intuniv.ab,ti.	
38.	*clonidine/	
39.	Clonidine.ab,ti.	
40.	Kapvay.ab,ti.	
41.	viloxazine.ab,ti.	
42.	Qelbree.ab,ti.	
43.	8 or 9 or 10 or 11 or 12 or 13 or 14 or 15 or 16 or 17 or 18 or 19 or 20 or 21 or 22 or 23 or 24 or 25 or 26 or 27 or 28 or 29 or 30 or 31 or 32 or 33 or 34 or 35 or 36 or 37 or 38 or 39 or 40 or 41 or 42	
44.	exp rhinitis/ or exp chronic rhinitis/	
45.	Rhinitis.af.	
46.	exp nose obstruction/	
47.	Nasal Obstruction.af.	
48.	nose obstruction.af.	
49.	exp rhinopharyngitis/	
50.	Nasopharyngitis.af.	
51.	rhinopharyngitis.af.	
52.	exp upper respiratory tract infection/	
53.	"Upper Respiratory Tract Infection*".af.	
54.	"URTI*".af.	
55.	exp rhinorrhea/	
56.	"rhinorrh*".af.	
57.	"nasal symptom*".af.	
58.	"nasal*".af.	
59.	exp chronic sinusitis/ or exp acute sinusitis/ or exp sinusitis/	
60.	"sinusiti*".af.	
61.	sinonasal*.af.	
62.	44 or 45 or 46 or 47 or 48 or 49 or 50 or 51 or 52 or 53 or 54 or 55 or 56 or 57 or 58 or 59 or 60 or 61	
63.	7 and 43 and 62	

**Table 8:** Supplementary Appendix B - MEDLINE search strategy.

1.	attention deficit hyperactivity disorder/
2.	*Attention Deficit Disorder with Hyperactivity/
3.	adhd.ab,ti.
4.	Attention Deficit Hyperactivity Disorder.ab,ti.
5.	Attention Deficit Disorder with Hyperactivity.ab,ti.
6.	Attention Deficit Disorder.ab,ti.
7.	Hyperkinetic Syndrome.ab,ti.
8.	1 or 2 or 3 or 4 or 5 or 6 or 7
9.	*Central Nervous System Stimulants/
10.	*Methylphenidate/
11.	methylphenidate.ab,ti.
12.	Ritalin.ab,ti.
13.	Concerta.ab,ti.
14.	Medikinet.ab,ti.
15.	Equasym.ab,ti.
16.	Quillivant.ab,ti.
17.	Daytrana.ab,ti.
18.	Metadate.ab,ti.
19.	*Amphetamine/
20.	"amphetamine*".ab,ti.
21.	Adderall.ab,ti.
22.	Evekeo.ab,ti.
23.	Mydayis.ab,ti.
24.	*Dextroamphetamine/
25.	Dextroamphetamine.ab,ti.
26.	"dexamphetamine*".ab,ti.
27.	Dexedrine.ab,ti.
28.	*Lisdexamfetamine Dimesylate/
29.	Lisdexamphetamine.ab,ti.
30.	Vyvanse.ab,ti.
31.	*Atomoxetine Hydrochloride/
32.	Atomoxetine Hydrochloride.ab,ti.
33.	atomoxetine.ab,ti.
34.	Strattera.ab,ti.
35.	*Guanfacine/
36.	Guanfacine.ab,ti.
37.	Intuniv.ab,ti.
38.	*Clonidine/
39.	Clonidine.ab,ti.
40.	Kapvay.ab,ti.
41.	viloxazine.ab,ti.
42.	Qelbree.ab,ti.
43.	9 or 10 or 11 or 12 or 13 or 14 or 15 or 16 or 17 or 18 or 19 or 20 or 21 or 22 or 23 or 24 or 25 or 26 or 27 or 28 or 29 or 30 or 31 or 32 or 33 or 34 or 35 or 36 or 37 or 38 or 39 or 40 or 41 or 42
44.	exp Rhinitis/ or exp Rhinitis, Vasomotor/
45.	Rhinitis.af.
46.	exp Nasal Obstruction/
47.	Nasal Obstruction.af.
48.	nose obstruction.af.
49.	exp Nasopharyngitis/
50.	Nasopharyngitis.af.
51.	rhinopharyngitis.af.
52.	exp Respiratory Tract Infections/
53.	"Upper Respiratory Tract Infection*".af.
54.	"URTI*".af.
55.	exp Rhinorrhea/
56.	"rhinorrh*".af.
57.	"nasal symptom*".af.
58.	"nasal*".af.
59.	exp Sinusitis/
60.	"sinusiti*".af.
61.	sinonasal*.af.
62.	44 or 45 or 46 or 47 or 48 or 49 or 50 or 51 or 52 or 53 or 54 or 55 or 56 or 57 or 58 or 59 or 60 or 61
63.	8 and 43 and 62

**Table 9:** Supplementary Appendix C - COCHRANE search strategy.

#1	MeSH descriptor: [Attention Deficit Disorder with Hyperactivity] explode all trees
#2	("ADHD"):ti,ab,kw
#3	("attention deficit hyperactivity disorder"):ti,ab,kw
#4	(Attention Deficit Disorder with Hyperactivity):ti,ab,kw
#5	("attention deficit disorder"):ti,ab,kw
#6	("hyperkinetic syndrome"):ti,ab,kw
#7	#1 OR #2 OR #3 OR #4 OR #5 OR #6
#8	MeSH descriptor: [Central Nervous System Stimulants] explode all trees
#9	MeSH descriptor: [Methylphenidate] explode all trees
#10	(methylphenidate OR Ritalin OR Concerta OR Medikinet OR Equasym OR Quillivant OR Daytrana OR Metadate):ti,ab,kw
#11	MeSH descriptor: [Amphetamines] explode all trees
#12	(Adderall OR Evekeo OR Mydayis):ti,ab,kw
#13	MeSH descriptor: [Dextroamphetamine] explode all trees
#14	(Dextroamphetamine OR dexamphetamine OR Dexedrine):ti,ab,kw
#15	(Lisdexamphetamine OR Vyvanse OR Atomoxetine Hydrochloride OR atomoxetine OR Strattera):ti,ab,kw
#16	MeSH descriptor: [Atomoxetine Hydrochloride] explode all trees
#17	MeSH descriptor: [Guanfacine] explode all trees
#18	(Guanfacine OR Intuniv):ti,ab,kw
#19	MeSH descriptor: [Clonidine] explode all trees
#20	(Clonidine OR Kapvay OR viloxazine OR Qelbree):ti,ab,kw
#21	#8 OR #9 OR #10 OR #11 OR #12 OR #13 OR #14 OR #15 OR #16 OR #17 OR #18 OR #19 OR #20
#22	MeSH descriptor: [Rhinitis] explode all trees
#23	MeSH descriptor: [Nasal Obstruction] explode all trees
#24	MeSH descriptor: [Nasopharynx] explode all trees
#25	MeSH descriptor: [Respiratory Tract Infections] explode all trees
#26	MeSH descriptor: [Rhinosinusitis] explode all trees
#27	MeSH descriptor: [Rhinorrhea] explode all trees
#28	MeSH descriptor: [Sinusitis] explode all trees
#29	(rhinitis OR Nasal Obstruction OR nose obstruction OR Nasopharyngitis OR rhinopharyngitis OR Upper Respiratory Tract Infection OR URTI OR rhinorrhea OR nasal symptoms OR sinonasal OR sinusitis):ti,ab,kw
#30	#22 OR #23 OR #24 OR #25 OR #26 OR #27 OR #28 OR #29
#31	#7 AND #21 AND #30
